# Supplementary material for: End Sequence Analysis Toolkit (ESAT) expands the extractable information from single-cell RNA-seq data
Source: Genome Res. 2016 Oct;26(10):1397–410. doi: 10.1101/gr.207902.116 (PMC5052061; doi:10.1101/gr.207902.116)
Supplement: Supplemental Material [file supp_gr.207902.116_Supp_Materials.tar.gz › ESAT-esat_v0.1/doc/html/functions_t.html]

ESAT: Class Members


ESAT


- Main Page
- Related Pages
- Packages
- Classes
- Files

- Class List
- Class Index
- Class Hierarchy
- Class Members

- All
- Functions
- Variables
- Enumerator

- a
- b
- c
- d
- e
- f
- g
- h
- i
- j
- k
- l
- m
- n
- o
- p
- q
- r
- s
- t
- u
- v
- w
- z


All Classes Namespaces Files Functions Variables Enumerator Pages

Here is a list of all class members with links to the classes they belong to:

### - t -

- T7BASES
  : broad.pda.seq.rap.CountProbes
- T\_CODE
  : broad.core.multiplealignment.MultipleAlignment
- TAB
  : jaligner.util.Commons
- takeComplement()
  : broad.core.annotation.AnnotationReader< T extends GenomicAnnotation >
- takeIntersection()
  : broad.core.annotation.AnnotationReader< T extends GenomicAnnotation >
  , broad.core.annotation.BasicLightweightAnnotation
  , broad.core.annotation.LightweightGenomicAnnotation
  , broad.core.sequence.SequenceRegion
- takeUnion()
  : broad.core.annotation.BasicLightweightAnnotation
  , broad.core.annotation.LightweightGenomicAnnotation
  , broad.core.sequence.SequenceRegion
  , umms.core.annotation.Gene
- TARGET
  : broad.pda.seq.rap.BuildRatioNullDistribution
  , broad.pda.seq.rap.GenomeScoringProgram
  , broad.pda.seq.rap.RatioPermutationPeakCaller
  , broad.pda.seq.rap.rna.TranscriptomeScoringProgram
  , broad.pda.seq.rap.SkellamKLPeakCaller
- testGoodnessOfFit()
  : broad.core.math.EmpiricalDistribution
- threeFoldDegenerateCodons
  : broad.core.multiplealignment.AlignmentExtractor
- times()
  : broad.core.datastructures.MatrixWithHeaders
  , Jama.Matrix
- timesEquals()
  : Jama.Matrix
- toArray()
  : jaligner.Sequence
- toBED()
  : broad.core.annotation.BED
  , broad.core.sequence.SequenceRegion
  , umms.core.annotation.AbstractAnnotation
  , umms.core.annotation.Annotation
  , umms.core.annotation.Gene
- toBEDGraph()
  : broad.core.sequence.SequenceRegion
  , umms.core.annotation.AbstractAnnotation
  , umms.core.annotation.Annotation
- toBEDwithBedScore()
  : umms.core.annotation.Gene
- toCigar()
  : broad.core.sequence.SequenceRegion
  , umms.core.annotation.AbstractAnnotation
  , umms.core.annotation.Annotation
- toCollection()
  : broad.core.datastructures.IntervalTree< V >
- toConsensus()
  : broad.core.motif.PositionWeightColumn
- toConstituentIntroformMap()
  : broad.pda.annotation.BEDFileParser
- toConstituentIsoformMap()
  : broad.pda.annotation.BEDFileParser
- toCufflinksGTF()
  : umms.core.annotation.Gene
- toCufflinksString()
  : broad.core.annotation.GFF
- toDBFromAscii()
  : broad.core.sequence.FastaQualityBinaryIO
- toDoubleArray()
  : broad.core.math.Statistics
- toDoubleArrayList()
  : broad.core.math.Statistics
- toFasta()
  : broad.pda.seq.fastq.FastqSequence
- toFastq()
  : broad.pda.seq.fastq.FastqSequence
- toFileName()
  : broad.pda.datastructures.Alignments
- toFilledInGene()
  : broad.pda.seq.graph.Path
- toGene()
  : broad.pda.seq.graph.Path
- toGTF()
  : umms.core.annotation.Gene
- toList()
  : broad.core.datastructures.IntervalTree< V >
  , umms.core.annotation.AnnotationList< T extends Annotation >
- toMap()
  : broad.core.datastructures.MatrixWithHeaders
  , broad.pda.annotation.BEDFileParser
- toMultipleAlignment()
  : broad.core.multiplealignment.MAFAlignment
- toNanostring()
  : broad.pda.rnai.RNAiGeneAnnotation
- toRefSeq()
  : umms.core.annotation.Gene
- toRNAi()
  : broad.pda.rnai.RNAiGeneAnnotation
- toSAM()
  : umms.core.annotation.Gene
- toSAMRecord()
  : umms.core.alignment.AbstractPairedEndAlignment
  , umms.core.alignment.Alignment
  , umms.core.alignment.SingleEndAlignment
- toShortBED()
  : broad.core.sequence.SequenceRegion
  , umms.core.annotation.AbstractAnnotation
  , umms.core.annotation.Annotation
- toShortString()
  : broad.core.annotation.BED
  , broad.core.annotation.ShortBED
- toString()
  : broad.core.annotation.BasicGenomicAnnotation
  , broad.core.annotation.BasicLightweightAnnotation
  , broad.core.annotation.BasicTwoSubjectAnnotation
  , broad.core.annotation.BED
  , broad.core.annotation.GFF
  , broad.core.annotation.RepeatMaskerAnnotation
  , broad.core.annotation.ShortBED
  , broad.core.motif.PositionWeightColumn
  , broad.core.motif.SequenceMotif
  , broad.core.sequence.SequenceRegion
  , broad.core.siphy.EvolutionaryModelParameters
  , broad.pda.datastructures.Alignments
  , broad.pda.enrichment.EnrichmentMetric
  , broad.pda.gene.GeneWithIsoforms
  , broad.pda.geneexpression.clustering.Cluster
  , broad.pda.rnai.RNAiGeneAnnotation
  , broad.pda.seq.fastq.FastqSequence
  , broad.pda.seq.graph.Path
  , broad.pda.seq.rap.SkellamScore
  , jaligner.Alignment
  , jaligner.Sequence
  , Jama.Matrix
  , umms.core.alignment.AbstractPairedEndAlignment
  , umms.core.alignment.SingleEndAlignment
  , umms.core.annotation.Annotation.Strand
  , umms.core.annotation.BasicAnnotation
  , umms.core.annotation.CompoundInterval
  , umms.core.annotation.Gene
  , umms.core.annotation.SingleInterval
  , umms.core.model.score.AbstractListScore
  , umms.core.model.score.BasicScanStatisticScore
  , umms.core.model.score.CountScore
  , umms.core.model.score.NewRatioScore
  , umms.core.model.score.RatioScore
  , umms.core.model.score.ScanStatisticScore
  , umms.core.model.score.WindowAllScore
- toStringForWriting()
  : broad.core.annotation.AnnotationReader< T extends GenomicAnnotation >
- toStringNum()
  : broad.pda.datastructures.Alignments
- toStringWithScores()
  : broad.pda.datastructures.Alignments
- totalCount
  : broad.pda.enrichment.EnrichmentMetric
- toUCSC()
  : broad.core.annotation.BasicLightweightAnnotation
  , broad.core.annotation.LightweightGenomicAnnotation
  , broad.core.sequence.SequenceRegion
  , broad.pda.datastructures.Alignments
  , umms.core.annotation.AbstractAnnotation
  , umms.core.annotation.Annotation
  , umms.core.annotation.Gene
- toUniqueSortedList()
  : broad.core.math.Statistics
- toWIGString()
  : broad.core.annotation.BED
  , broad.core.annotation.ShortBED
- trace()
  : Jama.Matrix
- TracebackNode()
  : jaligner.TracebackNode
- track()
  : broad.core.annotation.AnnotationHandler
  , broad.pda.datastructures.LocationAwareMatrix
- TranscribedRegions()
  : broad.core.sequence.TranscribedRegions
- TranscribedSequenceComposition()
  : broad.core.sequence.TranscribedSequenceComposition
- TranscriptInGenomicSpace()
  : umms.core.coordinatesystem.TranscriptInGenomicSpace
- TRANSCRIPTION\_READ
  : broad.pda.seq.rap.rna.TranscriptomeCommandLineProgram
- transcriptomeSpace
  : broad.pda.seq.protection.TwoSampleScanSkellamPeakCaller
- TranscriptomeSpace()
  : umms.core.coordinatesystem.TranscriptomeSpace
- transcriptsNullModel()
  : broad.pda.gene.transcriptsNullModel
- transcriptToGenomicPosition()
  : umms.core.annotation.Gene
- translocate()
  : broad.pda.feature.genome.DirectoryInstalledGenomeAssembly
- transpose()
  : Jama.Matrix
- TreeScaler()
  : broad.core.siphy.TreeScaler
- TreeScalerIO()
  : broad.core.siphy.TreeScalerIO
- trim()
  : broad.core.sequence.SequenceRegion
- TRIM
  : broad.pda.seq.rap.RatioPermutationPeakCaller
- trim()
  : umms.core.annotation.AbstractAnnotation
  , umms.core.annotation.Annotation
- trimAbsolute()
  : umms.core.annotation.Gene
- trimBeginning()
  : broad.pda.seq.fastq.FastqSequence
- trimByInformationContent()
  : broad.core.motif.PositionWeightMatrix
- trimEndBases()
  : broad.pda.seq.fastq.FastqSequence
- trimEnds()
  : broad.pda.seq.fastq.FastqSequence
- trimFirstNBPs()
  : broad.pda.seq.fastq.FastqSequence
- trimGene()
  : umms.core.annotation.Gene
- trimGeneEnds()
  : broad.pda.seq.segmentation.ContinuousDataAlignmentModel
- trimLinker()
  : broad.pda.seq.fastq.FastqSequence
- trimMaxContiguous()
  : broad.pda.seq.protection.SampleData
  , broad.pda.seq.segmentation.ContinuousDataAlignmentModel
- trimPeak()
  : broad.pda.seq.protection.TwoSampleScanSkellamPeakCaller
- trimPeakByQuantile
  : broad.pda.seq.protection.TwoSampleScanSkellamPeakCaller
- trimPolyA()
  : broad.pda.seq.fastq.FastqSequence
- trimStartBases()
  : broad.pda.seq.fastq.FastqSequence
- ts
  : broad.pda.seq.rap.rna.TranscriptomeCommandLineProgram
- tScoresByRow()
  : broad.core.datastructures.MatrixWithHeaders
- tstat()
  : broad.core.math.Statistics
- twoFoldDegenerateCodons
  : broad.core.multiplealignment.AlignmentExtractor


---

Generated on Fri Aug 15 2014 15:11:16 for ESAT by  

 1.8.7
